# Psychometric properties of the cultural mix coping inventory for stressful situations using physical education teachers: a multidimensional item response theory analysis

**DOI:** 10.1186/s40359-022-00916-3

**Published:** 2022-08-29

**Authors:** Frank Quansah, John Elvis Hagan, James Boadu Frimpong, Medina Srem-Sai, Edmond Kwesi Agormedah, Francis Ankomah

**Affiliations:** 1 Department of Educational Foundations, University of Education, Winneba, Ghana; 2 Department of Health, Physical Education and Recreation, University of Cape Coast, Cape Coast, Ghana; 3 Neurocognition and Action-Biomechanics-Research Group, Faculty of Psychology and Sports Science, Bielefeld University, Bielefeld, Germany; 4 Department of Health, Physical Education, Recreation and Sports, University of Education, P. O. Box 25, Winneba, Ghana; 5 Department of Business and Social Sciences Education, University of Cape Coast, Cape Coast, Ghana; 6 Department of Education and Psychology, University of Cape Coast, PMB Cape Coast, Cape Coast, Ghana; 7 Department of Education, SDA College of Education, P. O. Box AS 18, Asokore-Koforidua, Ghana

**Keywords:** Physical education, Teachers, Coping strategy, Multidimensional item response theory, Stress

## Abstract

The incidence of the COVID-19 pandemic heightened the levels of stress of not only students but for teachers, particularly physical education (PE) teachers. The reference to PE teachers is due to their role in engaging students in practical in-person lessons after the resumption of school. Previous literature has revealed that PE teachers exhibit significantly increased levels of anxiety, fear, tension and uncertainty that they could contract the virus during these lessons. Given this scenario, there is a growing need for identifying a suitable coping scale which can accurately measure coping strategies employed these teachers. This research assessed the psychometric properties of the 16-item coping inventory using a multidimensional item response theory approach. The study surveyed 484 PE teachers through the convenience sampling technique, after which the cultural mix coping instrument was administered to them. The findings of this study confirmed the 4-factor structure of the coping measure which is consistent with the original measure. Results further revealed that a modified 14-item compared to the original 16-item coping inventory was optimal in measuring coping strategies among PE teachers. The study concluded that the 14-item cultural mix coping inventory was appropriate, applicable and reproducible to the PE teachers’ population.

**Supplementary Information:**

The online version contains supplementary material available at 10.1186/s40359-022-00916-3.

## Introduction

Coping with stressful experiences in life has become a popular research construct and in applied practice. The adoption or use of coping plays a significant role in the psychological wellbeing of individuals because of its potential function as a protective mechanism against situations perceived as threatening or harmful [1]. Lazarus and Folkman [1] refer to coping as the set of continuously mutable cognitive-behavioural efforts to manage specific internal and/or external demands that are appraised by the individual as threatening and exceeding an individual’s resources. Previous research has already established a multiplicity of coping options used by individuals to manage a host of psychosocial stressors, stressing the usefulness and health impact of these measures through varied instruments with questionnaires dominating these measures [2–5]. These existing measures include coping instruments developed for athletes [6, 7], relatives/caregivers of schizophrenic patients [8, 9], children and adolescents [4, 10, 11], HIV patients and caregivers of children with cancer [12], students [3, 13], primary care patients [14], and the general population [15]. Given that numerous coping measures have been used by different population groups who experience varying levels of stress triggered by different stressors; therefore, these cohorts are unlikely to use the same mechanisms for coping. 

Despite these scholarly attempts toward the development of reliable coping instruments, existing measures have been subjected to many criticisms because of noteworthy limitations [4, 16]. Notably, previous coping measures required respondents to respond to hypothetical stressful situations in an instructional text [4]. The challenge is that these individuals might not have encountered such stressful situations and thus, their responses may not reflect their actual coping mechanisms in these stressful periods. Further, previous coping instruments have shown dissimilar factor structures and sub-categories which mirrored the sample for whose usage the inventory was planned, including poor psychometric properties, non-representative samples, and other weaknesses with the item-generation process [4, 16, 17]. Another major criticism surrounding existing coping instruments is the neglect of cultural diversity and identities [4, 15, 18]. Culture plays a distinct role in how individuals react to stimuli and explains the distinctions associated with how people adjust to stressful situations [18, 19]. According to some scholars, failure to develop coping instruments that reflect a particular cultural and social background would show a lack of practical applicability and utility, including validity threats of such measures to stressful situations and settings [4, 15, 20–22].

In response to the call for a more all-encompassing and cultural-sensitive coping instrument, Quansah et al. [23] developed and validated a cultural mix coping inventory that mirrors the context-specific identity using university students in Ghana. In addressing the criticism by DanIşman et al. [4], for example, Quansah et al. situated the stressful situation within the coronavirus pandemic (by including ''under the COVID-19 situation'' in the instruction) which made it possible for the specific coping behaviour(s) to be chosen. Other relevant contextual variable like religion was also considered in the development of their coping measure. Quansah and coworkers’ findings revealed a sixteen-item psychometrically sound coping inventory with a four-dimensional structure, namely: active coping, religious coping, behaviour disengagement, and emotional support [23]. However, the authors suggested that to guarantee the generalization and utility of the newly developed measure, further re-validation studies of the newly developed instrument are required with different samples. Though the cultural mix coping measure is a recently developed coping scale, it has been adopted for use in some empirical studies [24–26]. With this growing utilisation of the coping measure in recent research, there is the need to widen its applicability to diverse populations.

Several empirical research, including cross-occupational comparative studies, have demonstrated that teachers experience a high level of stress which negatively affects their health and wellbeing [27–29]. Although physical education (PE) teachers share numerous commonalities with other teachers in the profession [30], teaching PE further requires different considerations particularly because of the peculiar conditions in PE classes such as physical demands and field lessons [31, 32]. It is not surprising that recent pieces of literature have reported heightened levels of stress among PE teachers [33, 34]. The COVID-19 pandemic incidence intensified the levels of stress of PE teachers in Ghana [35], especially after the resumption of schools where PE teachers exhibited symptoms of anxiety, fear, tension and uncertainty that they could contract the virus [24, 36, 37] as they engaged students in practical in-person lessons. Based on the foregoing, this research applied the cultural mix coping measure to PE teachers who encountered stressful COVID-19 situations as they went about their formal duties in their respective schools.

Built on Quansah et al.’s study [23], the present research expands on the applicability of the 16-item cultural mix stress-coping scale to PE teachers through the lens of a multidimensional item response theory (MIRT) approach. The MIRT methodology was featured in this study for two main reasons. First, based on the findings from previous studies that the levels of stress among PE teachers differ depending on several factors like career experiences, cultural diversity, and other socio-demographic variables [38, 39], this research hypothesized that there are likely qualitative differences between the levels of ability to cope with stress in the PE teacher population. These perceived variations, consequently, make it problematic to measure coping ability by applying the same measurement items; hence, the need for the application of the MIRT method. Secondly, the study sought to also assess the item characteristics of the cultural mix coping measure which aimed at improving the scale by judging the adoption or rejection of items or adding new items based on the parameters of each item.

## Methods and materials

### Sample size and study participants

A total of 484 PE teachers were engaged in the study. The sample size was deemed sufficient based on the recommendations of Jiang et al. [40] that a sample size of 500 cases is sufficient to provide accurate parameter estimation in MIRT analysis. However, 16 of the respondents dropped out of the study resulting in a response rate of 96.8%. The sample was conveniently drawn from PE teachers of selected senior high schools in Ghana. The study was dominated by teachers who were 40 years and above (*n* = 214, 44.2%) while few (*n* = 12, 2.5%) of them were within the 20–24 years age category. The teachers were predominantly males (*n* = 357, 73.8%), with females being the minority (n = 127, 26.2%) group. Also, a greater proportion of the teachers were Christians (*n* = 448, 92%); Bachelor’s degree holders (*n* = 289, 59.7%) and had been teaching for at least 5 years (*n* = 300, 62%) (see Table 1).

**Table 1:** Demographic profile of the P.E. teachers

Variable	Levels	Frequency	Percent
Age	20–24	12	2.5
	25–29	60	12.4
	30–34	83	17.1
	35–39	115	23.8
	40 and above	214	44.2
Gender	Male	357	73.8
	Female	127	26.2
Religion	Christian	448	92.6
	Muslim	36	7.4
Education level	Certificate	18	3.7
	Diploma	57	11.8
	Bachelors	289	59.7
	Masters	120	24.8
Years of teaching	Less than 1 year	39	8.1
	1–2 years	54	11.2
	3–4 years	91	18.8
	Above 5 years	300	62.0

### Instrumentation

The study made use of the 16-item stress inventory developed by Quansah et al. [23]. The instrument had four subscales, namely, *active coping*, *religious coping*, *behaviour disengagement*, and *emotional support*. Each of the subscales had four items and was measured using four response options 1 = “*not adopted*”, 2 = “*somewhat* or *moderately adopted*”, 3 = “*much adopted*” and 4 = “*very much adopted*”. The preamble, “*Think about what strategies you usually resorted to when you were under stress induced by the COVID-19 pandemic*” preceded the items. The respondents were asked to provide their responses by indicating how they coped with the stressful COVID-19 situation (i.e., having in-person practical PE lessons) after the school resumption following school closure. Typical items on the instrument included “I concentrate my effort on doing something about it” for the active coping subscale, “I put my trust in God/object of worship” for religious coping, “I admit to myself that I can’t deal with the stressor and quit trying” for behaviour disengagement, and “I discuss how I feel about the stressor with someone” for emotional support. The coping inventory was reported to have sound psychometric properties. The omega reliability coefficients for the original version were 0.823, 0.812, 0.869, and 0.826 for active coping, religious coping, behaviour disengagement, and emotional support respectively [23]. The data from this study showed omega reliability estimates of 0.703, 0.835, 0.782, and 0.701 for active coping, religious coping, behaviour disengagement, and emotional support respectively.

### Procedure

The Institutional Review Board of the University of Cape Coast (UCCIRB) approved the research protocol with the reference number UCCIRB/EXT/2020/25. Respondents’ informed consent was obtained prior to the study. The contacts of the PE teachers were obtained from the Regional PE coordinators who were contacted directly. While some of the respondents opted for an online survey, others preferred a face-to-face administration. For the in-person administration, the COVID-19 protocols (e.g., wearing nose masks, hand washing, sanitizing hands and observing social distance in their classrooms) were strictly adhered to. With the authorization of the school authorities, prior arrangements were made with the teachers regarding the dates and time of data collection. The researchers distributed the questionnaires by hand to the respondents and retrieved them by hand. For the online administration, the electronic version of the instrument was shared with the respondents through their e-mails. The convenience sampling technique was employed to engage the teachers who were available and willing to respond to the instruments. Responding to the items on the instrument lasted for approximately 10–15 minutes. The respondents were assured of confidentiality, anonymity and freedom to opt out of the study at any point they deem necessary.

This research was part of ongoing research dubbed “Psychosocial Climate, Anxiety and Coping during COVID-19 pandemic in Secondary Schools”, which was carried out in the first quarter of 2021. It is instructive to state that some part of the project has been published on the COVID-19 awareness, anxiety and coping strategies of the general teacher population [24] as well as PE teachers’ COVID-19 knowledge, workplace safety perception, and anxiety response [35]. During this period, schools had resumed in-person teaching and learning activities after over 9 months of lockdown and strict restrictions. In a situation report by UNICEF [41] as of January 2021, Ghana had recorded 67,010 confirmed cases, 53,301, 416 deaths, and 5, 358 active cases. According to the UNICEF situation report, there was a prevailing upsurge in the number of cases at the time which compelled the government to announce further restrictions (https://www.unicef.org/media/92716/file/UNICEF-Ghana-COVID-19-Situation-Report-No.-14-1-31-January-2021.pdf). Within the same period, some secondary school students were also infected with the virus, with 142 cases as at February 2021 [42].

### Statistical methods and assumptions

The study utilized the graded responses model (GRM) [43], which is an extended form of the 2-parameter logistic model, which is used in instances where items are measured using an ordered scale [44]. The IRT PRO software (version 4.2) was used for the analysis [45]. The characteristics of the items were assessed based on two parameters: (1) discrimination estimate which examines the slope of the cumulative category response functions, and (2) difficulty estimate which assesses the value of the latent trait where the cumulative category response function equals 0.5. Because the scale option was 4, three difficulty parameters were computed, one for each threshold. These thresholds explain the value of the latent trait at which a respondent has a 50% chance of obtaining a score at or above a specific response category [46]. For the purposes of interpretation, slope parameter estimate less than 0.50 was deemed as poor, values from 0.50 to 1.0 was moderate, greater than  1.0 and equal to 1.50 was considered good, and greater than 1.50 was classified as excellent [47]. Item characteristic curves, item information curves, total information curve, and test characteristic curve were also used for interpreting the analysis [48].

For preliminary analyses, we conducted ordinal factor analysis (confirmatory factor analysis, CFA), assuming the 4-factor structure of the original coping measure, aimed at determining the applicability of the latent structure with the PE teacher population. The analysis was performed with the FACTOR computer program (Version 12.01.02) using the diagonally-weighted least squares (DWLS) method of estimation. The following model fit indices were obtained: χ^2^(62) = 132.575, *p* < 0.001; CMIN/*df* = 2.138; Comparative Fit Index (CFI) = 0.947, *CI *(0.936, 0.963); Standardized Root Mean Square Residual (SRMR) = 0.0481, *CI *(0.044, 0.050); Weighted Root Mean Square Residual (WRMR) = 0.0478, *CI *(0.044, 0.050); Root Mean Square Error of Approximation (RMSEA) = 0.038, *CI *(0.034, 0.048). The analysis confirmed the 4-factor structure of the original measure.

Further, the case adequacy for each response category per item was explored to assess how the respondents utilized the ordered response category of the scale. Satisfying this assumption is relevant because it has a direct effect on the accuracy of the parameters estimated [49]. The data retrieved showed that the number of cases falling into each response option was adequate, with most response options recording over 10% of observations (see Additional file 1: Appendix A). The local independence hypothesis was also met with majority of the item pairs showing low values (i.e., values less than 5) (see Additional file 1: Appendix B) indicating a low risk of systematic model fit problems [50, 51].

## Results

### Summary of preliminary analyses

The results from the CFA revealed that the specified model with a 4-factor structure and 16 items fit the data. The model fit indicators were sufficient and acceptable. Overall, the data from the PE teachers supported the 4-factor structure of the original cultural mix coping inventory, suggesting that the same factor structure was applicable to the PE teachers’ sample. Further, the reliability coefficients of the dimensions were greater than 0.70, indicating an adequate level of internal consistency.

### Model fit

Different model fit indices were reported. The loglikelihood fit statistics showed a value of 17,638.24. The reduced *M2* statistics for the multidimensional model was nonsignificant, *M2* = 17.32, *p* = 0.083. The RMSEA estimate was 0.032. The model fit indices supported the appropriateness of the model.

### Item parameter estimates

The results of the item parameter estimates are shown in Table 2.

**Table 2:** Graded model item parameter estimates, logit: *a*(*θ *– *b*)

Label	Items	*a*_*1*_* (se)*	*a*_*2*_*(se)*	*a*_*3*_*(se)*	*a*_*4*_*(se)*	*b*_1_	*s.e*	*b*_2_	*s.e*	*b*_3_	*s.e*
AC1	I concentrate my effort on doing something about it	.98 (0.13)	–	–	–	− 1.60	0.21	− 0.29	0.11	1.31	0.18
AC2	I take additional action to try to get rid of the problem	2.00 (0.24)	–	–	–	− 1.68	0.15	− 0.44	0.08	1.10	0.11
AC3	I take direct action to get around the stressor	1.67 (0.18)	–	–	–	− 1.34	0.13	− 0.41	0.08	1.73	0.15
AC4	I do what has to be done, one step at a time	2.45 (0.37)	–	–	–	− 1.99	0.17	− 0.45	0.07	0.39	0.08
RC1	I put my trust in God/object of worship	–	4.02 (0.59)	–	–	− 1.44	0.09	− 0.82	0.07	− 0.20	0.06
RC2	I seek help from my object of worship	–	3.06 (0.32)	–	–	− 1.28	0.09	− 0.75	0.07	− 0.04	0.07
RC3	I try to find comfort in my object of worship	–	2.37 (0.24)	–	–	− 1.58	0.12	− 0.66	0.07	− 0.08	0.07
RC4	I pray more than usual for my God to guard me	-	1.54 (0.16)	–	–	− 1.64	0.15	− 0.86	0.10	0.53	0.09
BD1	I admit to myself that I can’t deal with the stressor and quit trying	-	-	1.75 (0.18)	–	0.06	0.08	1.08	0.11	1.86	0.16
BD2	I just give up trying to reach my goal because of the stressor	–	–	3.03 (0.35)	–	0.03	0.07	0.67	0.07	1.62	0.12
BD3	I give up the attempt in dealing with the stressor	–	–	3.59 (0.51)	–	− 0.17	0.06	0.66	0.07	1.47	0.12
BD4	I reduce the amount of effort I’m putting into solving the problem	–	–	1.50 (0.16)	–	− 0.15	0.09	0.79	0.10	2.72	0.26
ESS1	I discuss how I feel about the stressor with someone	–	–	–	1.24 (0.14)	− 1.11	0.12	0.25	0.08	1.58	0.15
ESS2	I try to get emotional support from friends or relatives when dealing with the stressor	–	–	–	2.02 (0.28)	− 1.06	0.09	− 0.00	0.06	1.20	0.10
ESS3	I get sympathy and understanding from someone to reduce my fears about the problem	–	–	–	1.06 (0.12)	− 1.26	0.14	0.73	0.10	2.14	0.21
ESS4	I learn to live with the stressor	–	–	–	0.78 (0.10)	− 2.28	0.29	− 0.88	0.14	0.38	0.11

Marginal Reliability for Response Pattern Scores: 0.81; *se* standard error

*a*_*1*_ slope parameter for Active coping, *a*_*2*_ slope parameter for Religious coping, *a*_*3*_ slope parameter for behaviour disengagement coping, *a*_*4*_ slope parameter for emotional support

**Fig. 1:**
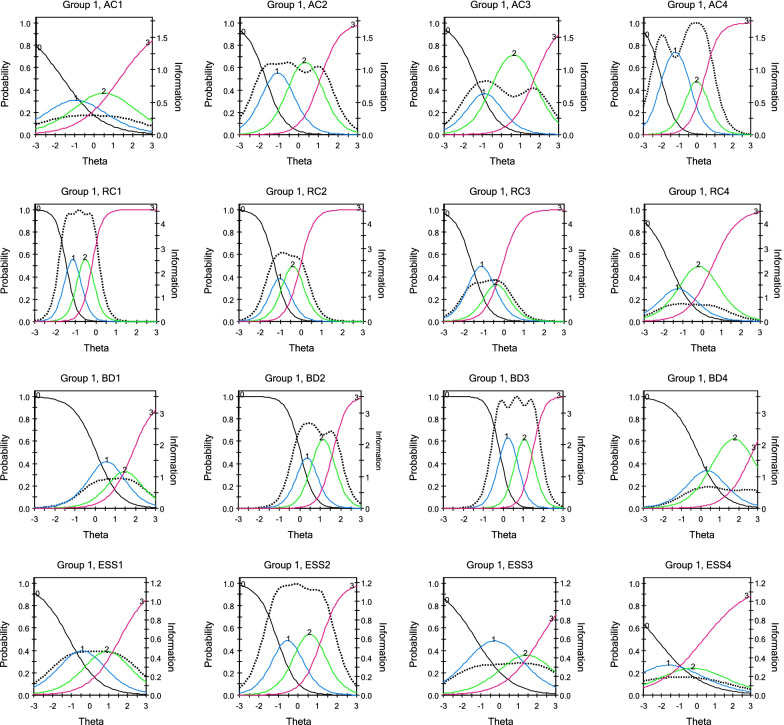
Item information and Characteristics Curve

The results revealed that the majority of the items had item discrimination parameters above 1.0, signifying good discrimination [44]. This implies that these items were able to discriminate across different ability levels, thereby, highlighting the fact that those who possessed more of the latent trait had higher scores on the items and vice versa. Some of the items had a low discrimination index, however. For the active coping domain, AC1 (“I concentrate my effort on doing something about it”, *a* = 0.98) had the least slope index and AC4 (“I do what has to be done, one step at a time”, *a* = 2.45) was the highest. Items RC4(“I pray more than usual for my God to guard me”, *a* = 1.54) and RC1 (“I put my trust in God/object of worship”, *a* = 4.02) showed the least and highest discrimination indices respectively for the religious coping dimension. For behaviour disengagement, BD4 (“I reduce the amount of effort I’m putting into solving the problem”, *a* = 1.50) showed the least slope index whereas BD3 (“I give up the attempt in dealing with the stressor”, *a* = 3.59) had the highest slope. Lastly, the emotional support subscale had ESS4 (“I learn to live with the stressor”, *a* = 0.78) showing the lowest discrimination index and ESS2(“I try to get emotional support from friends or relatives when dealing with the stressor”, *a* = 2.02) having the highest (see Table 2). The results also found that the difficulty threshold of all the items increased monotonically. Taking item RCI (“*I put my trust in God/object of worship*”), for example, the threshold parameters for *b*_*1*_, *b*_*2*_, and *b*_*3*_ are − 1.44, − 0.82, and − 0.20 respectively (see Table 2 and Fig. 1).

The item characteristic curves, as shown in Fig. 1, revealed that all the item thresholds increased monotonically. In addition, the item information curves depicted that every item, at least, had some empirical information they add to the measure of the construct. Item AC4 had the highest empirical information to the measure of active coping trait, whereas AC1 had the least empirical information to the measure of active coping dimension (see Fig. 1). Taking the religious coping construct, RC1 had the largest empirical information while item RC4 had the least. BD3 and ESS 2 showed the largest empirical information to the measure of behaviour disengagement coping and emotional support dimensions respectively (see Fig. 1). None of the items was found to be redundant, with each item providing unique information to the measurement of the construct.

### Test characteristic curve, total information function, standard error of estimate and marginal reliability

**Fig. 2:**
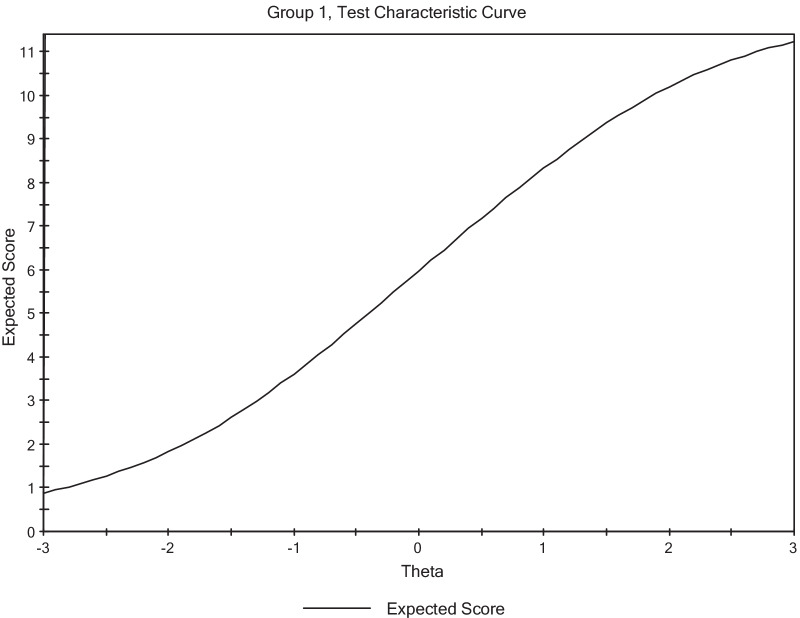
Test Characteristics Curve

Figure 2 shows the test characteristic curve which depicts how the latent trait level and expected scores of the participants are related.

The results from Fig. 2 show that there is generally, a positive relationship between the expected score and the ability scale. The test characteristic curve increases monotonically. The results indicate that the responses on the scale reflect that as the ability level of respondents increases, their expected score also increases.

**Fig. 3:**
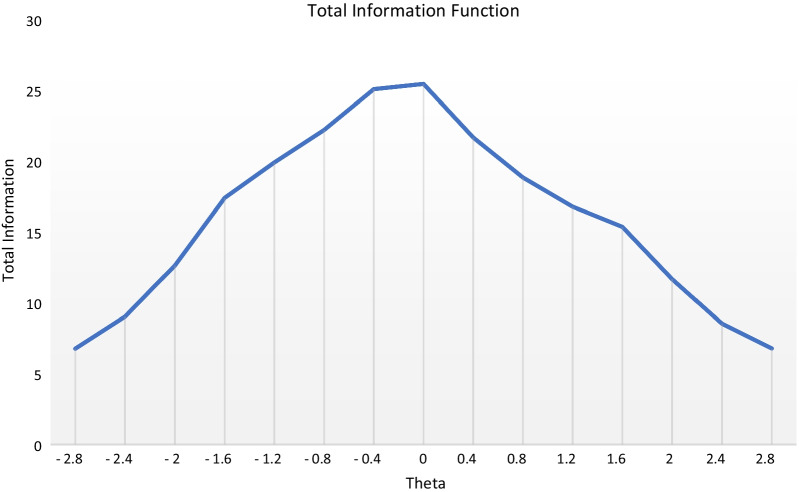
Total Information Function

Results of the total information function are shown in Fig. 3 and Table 3.

**Table 3:** Total information, standard error of estimate and reliability across θ values − 2.8 to 2.8

Theta Values	Total Information	Standard error of estimate	Marginal Reliability
− 2.8	6.74	0.80	0.851
− 2.4	8.99	0.71	0.889
− 2	12.59	0.63	0.921
− 1.6	17.35	0.57	0.942
− 1.2	19.84	0.52	0.950
− 0.8	22.13	0.47	0.955
− 0.4	25.00	0.43	0.960
0	25.37	0.43	0.961
0.4	21.60	0.45	0.954
0.8	18.78	0.49	0.947
1.2	16.73	0.54	0.940
1.6	15.31	0.59	0.935
2	11.63	0.65	0.914
2.4	8.50	0.73	0.882
2.8	6.76	0.79	0.852

The total information function provides relevant information as a function of location on the latent trait continuum and thus, tells how the entire scale works [52]. The test information function offers a relatively uniform information function between the range of − 0.4 and 0, which has a marginal reliability estimate of approximately 0.96 and a standard error (of estimate) of 0.43 as displayed in Fig. 3 and Table 3. However, the test provides its maximum information at an ability level of 0. Ability levels outside this range showed decreasing reliability and increasing standard error of the estimate.

## Discussion

The study assessed the psychometric properties of the 16-item coping inventory through MIRT analysis among PE teachers as they respond to stressful situations during the COVID-19 pandemic. Particularly, we assessed the item characteristics of the cultural mix coping measure aimed at improving the scale by judging the adoption or rejection of items or adding new items based on the parameters of each item. This validation study confirmed a similar factor structure as the original coping measure by Quansah et al. [23]. Further, the item characteristics of the items were promising, except for a few ones which failed to demonstrate optimum levels of functioning in measuring PE teachers’ ability to cope with stressful situations.

Among the items in the active coping dimension, AC1 provides the least information for all levels of active coping. However, it can best be used to identify individuals with varying levels of active coping. This outcome implies that AC1 is most useful in surveying people across all levels of active coping, but cannot efficiently differentiate among the active coping levels. AC2 and AC3 provide more useful information for persons who are a little close to or above the average level of active coping and as well differentiate among persons with coping in that spectrum. In the case of item AC4, though it provides the most information to the scale, it functions best for individuals with average or below-average levels of active coping with stress. By implication, items AC2, AC3, and AC4 may not be good items for the general population, but for persons with specific active coping levels.

For the religious coping dimension, RC1 offered the highest information. This item is considered most useful, working best for persons with moderately low (one standard deviation below average) ability in performing religious coping for stressful situations. Items RC2 and RC3 relatively could provide as much information as RC1; they similarly work better for moderately low to average religious coping. Item RC4, on the other hand, provides the least information function and functions best for moderately low to average religious coping abilities. Regarding the behaviour disengagement dimension, items BD3 and BD2, respectively, provided the most information for persons with abilities moderately above average in the adoption of behaviour disengagement as a coping mechanism. As such, these items could effectively discriminate for a person with such levels of behaviour disengagement. Items BD1 and BD4 provided relatively more information for persons with varying levels of behaviour disengagement, particularly, those in the above-average category. Items ESS1, ESS3, and ESS4 were more suitable in the provision of information for individuals across all levels of emotional support relative to item ESS2 which was informative for persons with moderately low use of emotional support. The item ESS2 could best differentiate among PE teachers with moderately low or average ability in terms of their social support. In all, items AC1 and ESS4, appear to provide much information for general populations in terms of screening, since they could not differentiate between coping levels with stressful situations.

Although all the items showed good indicators of the coping measure and for their respective domains, two items were not of high quality. The poorest of them was ESS4 (i.e., “*I learn to live with the stressor*”) which is captured under the emotional support sub-dimension. This finding suggests that the item did not function optimally in terms of contributing to the scaling of PE teachers into those who adopt high levels of emotional support and those who do not. This result may suggest that the interpretation provided by the item “*I learn to live with the stressor*” may contribute to the entire measure of coping but not to the emotional support domain. The statement “*I learn to live with the stressor*”, though reflects developing an internal mechanism for emotionally dealing with the stressor, may also explain a form of emotional disengagement when the individual has failed to internalize the ability to survive while living with the stressor [53]. This perspective was also stressed by Parkinson and McBain [54] that emotions play a critical role in behaviour disengagement and these possibilities may affect the functioning of the item “*I learn to live with the stressor*” among the PE teachers.

A key point to note from the findings is that the test information function was relatively stable between − 0.4 and 0, with ability being estimated close to the centre of the ability scale. This result suggests that the estimation of coping strategies tends to be relatively less precise at the extreme ends of the ability scale. The distribution of the total information function in this study reflects a promising feature of the coping measure and appears suitable for measuring PE teachers' ability to coping with stress for two reasons. First, every person adopts varied forms of coping strategies and as such, it is necessary to measure ability (i.e., coping) with sufficient precision at ability levels close to the ability used to separate persons who will utilize a specific coping strategy (e.g., active coping) from those who do not. Secondly, the identification of persons who adopt dysfunctional coping mechanisms for counselling or therapeutic purposes requires the coping inventory to have varying amounts of information for different ranges of ability levels [49]. Hence, the test information function does well in terms of estimating the coping mechanisms of PE teachers over a range of ability levels. Based on these arguments, the item information function for specific items suggests that items AC1 and ESS4 are not suitable for measuring coping strategies for PE teachers with varying levels of ability to cope with stress.

Generally, the re-validation of the cultural mix coping inventory for stressful situations among P.E teachers revealed promising results and the findings support the initial development and validation of the original scale by Quansah et al. [23]. Even though different estimation procedures were employed for the original validation and in this present study, the instrument showed similar levels of functioning of the inventory in terms of accurately estimating the coping strategies adopted by the study participants. Thus, the cultural mix coping inventory is appropriate for use for samples involving both students and teachers. Perhaps, the findings of this study support the use of the coping inventory among teachers due to a number of reasons. First, the COVID-19 pandemic presented stressful situations to everyone around the globe, and in the school setting, where students and teachers suffered the most [32, 35, 55, 56]. Furthermore, teachers and students are inseparable within the school context such that whatever affects the teacher also affects the student. For example, the utilization of unfamiliar pedagogical skills by teachers has the propensity of affecting students learning [57], especially in the era of COVID-19 pandemic. For PE teachers, this stressful situation appeared to be heightened after school resumption during the pandemic due to practical PE lessons [36]. These compelling reasons may have accounted for this case where the coping inventory was found to have functioned equivalently with both students’ and teachers’ samples.

### Implications for theory and practice

The findings of this research provide a basis for the continuous discourse on the validity and reproducibility of the recently developed coping inventory. The findings support the use of the instrument among students and teachers. Thus, researchers in psychology-related fields can use this inventory in studies that seeks to measure coping as a variable. A novel finding is that all the items on the coping inventory do not contribute the same amount of information to the measure. Similarly, the utility of the items varies for various levels of stress coping for different persons. Therefore, users of this inventory should be mindful of the purpose for which the inventory is used. Essentially, items ACI and ESS4 are not useful in differentiating PE teachers' levels of ability to cope with stressful situations. Because the instrument provides a precise measure of coping strategies adopted by students (16 items) and teachers (14 items), it can be used to scale individuals into different coping strategies they adopt most. Based on this dichotomy, those who employ dysfunctional coping strategies using items BD1, BD2, BD3, and BD4 (i.e., these items evaluated participants’ behaviours which reflect making no effort or completely reducing their efforts towards dealing with perceived stressors) can be identified and helped accordingly by rolling out to them some carefully designed interventions. This suggestion is very essential since dysfunctional coping is associated with poor mental health and psychological wellbeing [58–60]. The coping measure can as well be adopted in experimental studies.

### Limitations and future studies

The recommended minimum sample size for MIRT analysis is 500 cases to ensure precise estimation. In this study, the sample size was not up to the recommended sample (even though close) and this may compromise the power of the MIRT analysis conducted, thereby affecting the precision of the parameter estimates. This may also explain why some of the items did not function optimally. Furthermore, this validation was conducted with PE teachers and this may limit the generalization of the findings. It is recommended, therefore, that future studies should use a large sample size (over 500). Also, upcoming scholars are encouraged to validate this coping measure using the general teacher population to understand the applicability of the instrument across the teacher populace.

## Conclusion

The study evaluated the psychometric properties of the 16-item cultural mix coping inventory using a multidimensional item response theory (MIRT) approach. Our findings showed that the 14-item cultural mix coping inventory is appropriate, applicable and reproducible to the teachers’ population. Therefore, the coping instrument is recommended for use among teachers. This notwithstanding, the study encourages scholars within the field of psychology to continue the re-validation of the coping measure to widen its applicability.

## Supplementary Information

**Additional file 1**. Appendices A and B. The Appendix A presents the frequency and percentage counts for the responses provided on all the items. Appendix B presents the marginal fit (X2) and standardized LD X2 Statistics (local independence) for the items.

## Data Availability

The data and materials are available upon request through the corresponding author.
