# Supplementary material for: Psychometric properties of the cultural mix coping inventory for stressful situations using physical education teachers: a multidimensional item response theory analysis
Source: BMC Psychol. 2022 Aug 29;10:209. doi: 10.1186/s40359-022-00916-3 (PMC9422939; doi:10.1186/s40359-022-00916-3)
Supplement: Supplementary file 1 — Additional file 1. Appendices A and B. The Appendix A presents the frequency and percentage counts for the responses provided on all the items. Appendix B presents the marginal fit (X2) and standardized LD X2 Statistics (local independence) for the items. [file 40359_2022_916_MOESM1_ESM.docx]

**Appendix A**

Frequency and Percentage counts

| **S/N** | **Items** | not adopted | somewhat | much adopted | Very much adopted |
| --- | --- | --- | --- | --- | --- |
| **----** | ACTIVE COPING |  |  |  |  |
| AC1 | I concentrate my effort on doing something about it | 102(21.1) | 116(24.0) | 145(30.0) | 121(25.0) |
| AC2 | I take additional action to try to get rid of the problem | 46(9.5) | 135(27.9) | 209(43.2) | 94(19.4) |
| AC3 | I take direct action to get around the stressor | 84(17.4) | 107(22.1) | 238(49.2) | 55(11.4) |
| AC4 | I do what has to be done, one step at a time | 24(5.0) | 150(31.0) | 135(27.9) | 175(36.2) |
| -- | RELIGIOUS COPING |  |  |  |  |
| RC1 | I put my trust in God/object of worship | 42(9.7) | 74(15.3) | 103(21.1) | 266(55.0) |
| RC2 | I seek help from my object of worship | 66(13.6) | 72(14.9) | 107(22.1) | 239(49.4) |
| RC3 | I try to find comfort in my object of worship | 51(10.5) | 106(21.9) | 79(16.3) | 248(51.2) |
| RC4 | I pray more than usual for my God to guard me | 66(13.6) | 70(14.5) | 176(36.4) | 172(35.5) |
| -- | BEHAVIOURAL DISENGAGEMENT COPING |  |  |  |  |
| BD1 | I admit to myself that I can’t deal with the stressor and quit trying | 241(49.8) | 134(27.7) | 67(13.8) | 42(8.7) |
| BD2 | I just give up trying to reach my goal because of the stressor | 235(48.6) | 101(20.9) | 115(223.8) | 33(6.8) |
| BD3 | I give up the attempt in dealing with the stressor | 205(42.4) | 131(27.1) | 109(22.5) | 39(8.1) |
| BD4 | I reduce the amount of effort I’m putting into solving the problem | 215(44.4) | 121(25.0) | 130(26.9) | 18(3.7) |
| -- | EMOTIONAL SUPPORT |  |  |  |  |
| ES1 | I discuss how I feel about the stressor with someone | 93(19.2) | 137(28.3) | 176(36.4) | 78(16.1) |
| ES2 | I try to get emotional support from friends or relatives when dealing with the stressor | 102(21.1) | 134(27.7) | 172(35.5) | 76(15.7) |
| ES3 | I get sympathy and understanding from someone to reduce my fears about the problem | 114(23.6) | 135(27.9) | 157(32.4) | 78(16.1) |
| ES4 | I learn to live with the stressor | 24(5.0) | 96(19.8) | 158(32.6) | 206(42.6) |

**Appendix B**

Local independence

**Marginal fit (*X*^2^) and Standardized LD *X*^2^ Statistics for Group 1**[**(Back to TOC)**](file:///D:\MANUSCRIPTS\Covid%20&%20Work%20Climate\Coping_Valiation\MIRT%20Analysis\Psy_paper_Validation.Test1-irt.htm#home)

| Item | Label | 1 | 2 | 3 | 4 | 5 | 6 | 7 | 8 | 9 | 10 | 11 | 12 | 13 | 14 | 15 |
| --- | --- | --- | --- | --- | --- | --- | --- | --- | --- | --- | --- | --- | --- | --- | --- | --- |
| 1 | AC1 |  |  |  |  |  |  |  |  |  |  |  |  |  |  |  |
| 2 | AC2 | 0.4 |  |  |  |  |  |  |  |  |  |  |  |  |  |  |
| 3 | AC3 | 5.0 | 4.1 |  |  |  |  |  |  |  |  |  |  |  |  |  |
| 4 | AC4 | 2.5 | 3.5 | 4.6 |  |  |  |  |  |  |  |  |  |  |  |  |
| 5 | RC1 | 3.0 | 2.7 | 1.2 | 1.2 |  |  |  |  |  |  |  |  |  |  |  |
| 6 | RC2 | 1.0 | 1.1 | 1.3 | 4.2 | 4.9 |  |  |  |  |  |  |  |  |  |  |
| 7 | RC3 | 1.9 | 1.3 | 8.6 | 1.7 | 2.6 | 2.1 |  |  |  |  |  |  |  |  |  |
| 8 | RC4 | 1.2 | 1.7 | 1.3 | 6.7 | 9.5 | 1.3 | 2.1 |  |  |  |  |  |  |  |  |
| 9 | BD1 | 1.1 | 1.1 | 1.9 | 1.7 | 1.1 | 2.1 | 1.5 | 1.4 |  |  |  |  |  |  |  |
| 10 | BD2 | 1.3 | 1.5 | 1.8 | 7.8 | 1.9 | 1.3 | 3.1 | 1.1 | 1.1 |  |  |  |  |  |  |
| 11 | BD3 | 2.9 | 3.0 | 2.9 | 1.4 | 2.3 | 1.1 | 1.5 | 2.8 | 4.7 | 6.6 |  |  |  |  |  |
| 12 | BD4 | 0.7 | 1.3 | 2.6 | 1.4 | 1.0 | 1.9 | 2.1 | 1.5 | 1.3 | 4.7 | 6.2 |  |  |  |  |
| 13 | ESS1 | 2.7 | 1.1 | 5.3 | 1.3 | 1.6 | 2.7 | 1.7 | 7.7 | 2.7 | 2.8 | 2.0 | 8.7 |  |  |  |
| 14 | ESS2 | 1.9 | 3.0 | 7.8 | 4.5 | 2.8 | 1.6 | 1.5 | 1.5 | 1.5 | 1.3 | 2.6 | 3.1 | 4.3 |  |  |
| 15 | ESS3 | 2.4 | 1.0 | 5.2 | 5.0 | 1.8 | 2.4 | 1.6 | 1.8 | 1.1 | 1.7 | 4.2 | 2.4 | 3.5 | 2.1 |  |
| 16 | ESS4 | 1.3 | 5.2 | 7.7 | 2.4 | 1.6 | 1.7 | 1.6 | 1.9 | 1.7 | 1.2 | 2.1 | 8.8 | 1.6 | 1.2 | 3.9 |
